# The Influence of Reactive Ion Etching Chemistry on the Initial Resistance and Cycling Stability of Line-Type (Bridge) Phase-Change Memory Devices

**DOI:** 10.3390/ma18204681

**Published:** 2025-10-12

**Authors:** Abbas Espiari, Henriette Padberg, Alexander Kiehn, Kristoffer Schnieders, Jiayuan Zhang, Gregor Mussler, Stefan Wiefels, Abdur Rehman Jalil, Detlev Grützmacher

**Affiliations:** 1 Peter Grünberg Institute (PGI-10), Forschungszentrum Jülich GmbH, 52425 Jülich, Germany; a.kiehn@fz-juelich.de (A.K.); g.mussler@fz-juelich.de (G.M.); a.jalil@fz-juelich.de (A.R.J.); d.gruetzmacher@fz-juelich.de (D.G.); 2 Peter Grünberg Institute (PGI-7), Forschungszentrum Jülich GmbH, 52425 Jülich, Germany; henriette.padberg@rwth-aachen.de (H.P.); k.schnieders@fz-juelich.de (K.S.); s.wiefels@fz-juelich.de (S.W.); 3 Peter Grünberg Institute (PGI-9), Forschungszentrum Jülich GmbH, 52425 Jülich, Germany; ji.zhang@fz-juelich.de

**Keywords:** PCM, neuromorphic computing, phase-change materials, process optimization, reactive ion etching, endurance

## Abstract

Phase-change memory (PCM) is a promising candidate for in-memory computation and neuromorphic computing due to its high endurance, low cycle-to-cycle variability, and low read noise. However, among other factors, its performance strongly depends on the post-lithography fabrication steps. This study examines the impact of reactive ion etching (RIE) on PCM device performance by evaluating different etching gas mixtures, CHF_3_:O_2_, H_2_:Ar, and Ar, and determining their impact on key device characteristics, particularly initial resistance and cycling stability. The present study demonstrates that a two-step etching approach in which the capping layer is first removed using H_2_:Ar and the underlying GST layer is subsequently etched using physical Ar sputtering ensures stable and reliable PCM operation. In contrast, chemically reactive gases negatively impact the initial resistance, cycling stability, and device lifetime, likely due to alterations in the material composition. For the cycling stability evaluation, an advanced measurement algorithm utilizing the aixMATRIX setup by aixACCT Systems is employed. This algorithm enables automated testing, dynamically adjusting biasing parameters based on cell responses, ensuring a stable ON/OFF ratio and high-throughput characterization.

## 1. Introduction

Phase-change memory (PCM) is a leading candidate for next-generation nonvolatile memory due to its scalability, high endurance, low read noise, and fast-switching capabilities [1,2,3]. These features make PCM particularly attractive for neuromorphic computing and in-memory processing architectures, which aim to overcome the limitations of traditional von Neumann systems by reducing data transfer and energy consumption [4,5,6,7,8,9].

PCM devices function by utilizing resistive (Joule) heating to reversibly switch the phase-change material between an amorphous high-resistance state (HRS), achieved through a RESET operation, and a crystalline low-resistance state (LRS), established during a SET operation. The SET and RESET operations differ in pulse amplitude, duration, and cooling rate, determining whether the cell transitions to the crystalline or amorphous state [10]. A well-known class of phase-change materials can be found within the ternary Ge-Sb-Te diagram [11], including compounds such as Ge_2_Sb_2_Te_5_ (GST-225) or materials such as (Ag,In)-doped Sb_2_Te (AIST) [12,13,14,15,16,17].

Reliable device characteristics are essential for integrating PCMs into large-scale arrays, in-memory storage, and neuromorphic circuits. Apart from low variability, stable cycling performance is paramount for these applications, where repeated programming cycles are essential [18,19]. Cycling stability is not only a critical reliability factor but also a valuable metric for evaluating fabrication quality and optimizing fabrication steps.

The device performance heavily depends on the design of the PCM cell. The widely used contact-minimized, heater-based mushroom-cell design [10,20] shows promising characteristics but requires high power, making integration into standard-logic complementary metal–oxide–semiconductor (CMOS) operations more challenging [10,11].

To overcome these challenges, several volume-minimized structures have been developed [21]. Among them, the line cell structure [10,22] has emerged as a promising design in which the phase-change layer is patterned into a narrow line-shaped mesa (bridge) known as the active region. The active region is terminated on both sides by wider pads made of the same phase-change material, each connected to a bottom electrode. Since the active region reaches high temperatures during the SET and RESET operations, increasing the separation between this region and the metallic electrodes by using wide pads helps mitigate unwanted chemical reactions with the bottom electrodes. This self-heating, volume-minimized line cell design features a simple geometry and reduced active volume, which lowers power consumption. It also enables systematic investigation of how different reactive ion etching gases affect device functionality. The latter point is due to the elimination of other sources of interference such as unwanted reactions with the electrodes or loss of adhesion to the electrodes or heater at higher temperatures [10]. In previous studies, various RIE processes have been explored to structure phase-change materials, particularly GeTe and Sn-doped Ge_2_Sb_2_Te_5_, using gas mixtures such as CHF_3_:O_2_ and BCl_3_:Ar, with a focus on optimizing etch rates, selectivity, and surface morphology [23,24,25,26]. It has been shown that the incorporation of oxygen in CHF_3_-based etching improves etch selectivity but also leads to the formation of nonvolatile residues such as GeF_x_, GeO_x_, TeF_x_, and TeO_x_ [23,24]. Similarly, the use of BCl_3_:Ar plasma etching has demonstrated enhanced surface smoothness and a stable stoichiometric ratio, although it still leaves behind halogenated byproducts that necessitate additional cleaning steps [26].

In contrast to previous approaches, this work investigates the impact of different plasma etching environments, namely, CHF_3_:O_2_, H_2_:Ar, and pure Ar, on the fabrication of line-type PCM devices. Similar to previous studies, CHF_3_:O_2_ provides a chemically reactive reference case, but is known to leave nonvolatile residues on the etched GST surfaces. H_2_:Ar is included as an intermediate approach because such plasmas are commonly employed for etching Zn-based II–VI compounds, including ZnS, ZnSe, and ZnMgSSe [27], which are chemically similar to the ZnS:SiO_2_ capping layer used in our devices. In this environment, hydrogen reduces surface oxides, while argon contributes physical sputtering. Pure Ar plasma, in turn, serves as a purely physical etching environment, enabling residue-free structuring. This study also examines how these environments influence initial device resistance and the stability of cycling parameters, such as the SET and RESET voltages. This study uniquely integrates an automated measurement system (aixMATRIX) to dynamically adjust biasing conditions based on real-time device response, enabling a more systematic cycling analysis. The results demonstrate that, in contrast to chemically reactive plasma etching, a two-step approach in which the ZnS:SiO_2_ capping layer is removed via H_2_:Ar and the GST layer is subsequently etched by physical Ar sputtering ensures residue-free fabrication and stable PCM operation. This work presents an optimized etching approach for the fabrication of Ge-Sb-Te-based PCM devices, ensuring both high cycling stability and reproducibility, making it highly relevant for neuromorphic and in-memory computing applications.

## 2. Materials and Methods

### 2.1. Sample Preparation

**Figure 1:**
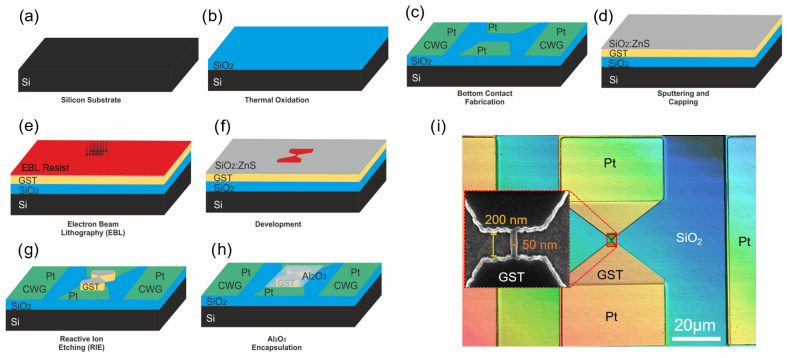
(**a**–**h**) Process flow of sample fabrication using Ge-Sb-Te compounds as the phase-change material. The design includes a coplanar waveguide (CPW) structure which is not utilized in the presented measurements. (**i**) An optical microscope image of a single line cell with an inset showing a scanning electron microscope (SEM) image of a line cell with $w_{\mathrm{bridge}}=50$ nm and $L_{\mathrm{bridge}}=200$ nm.

The fabrication flow of a PCM line cell is illustrated in Figure 1a–h. Initially, a silicon (Si) wafer is thermally oxidized to a thickness of 500 nm to reduce thermal dissipation and minimize leakage current. The wafer is then coated with a negative-tone optical resist (AZ nLOF 2020, Merck KGaA, Darmstadt, Germany), and subsequently optical lithography is performed. After development in AZ MIF 326 (Merck KGaA, Darmstadt, Germany), the electrodes are fabricated by electron beam evaporation of a 10 nm titanium (Ti) adhesion layer, followed by 20 nm platinum (Pt), and finalized with a lift-off process. Following this, the different antimony-based phase-change materials are deposited at room temperature via magnetron sputtering, a physical vapor deposition (PVD) technique widely used for producing uniform thin films with good control over morphology and composition [28].

These include AIST and germanium–antimony–telluride (GST) alloys with different stoichiometries: Ge_1_Sb_2_Te_4_ (GST-124), Ge_2_Sb_2_Te_5_ (GST-225), and Ge_1_Sb_4_Te_7_ (GST-147). Stoichiometric targets of Ag_4_In_3_Sb_67_Te_26_ and the mentioned GST compositions are employed to sputter layers with a thickness of 25 nm. The deposition is carried out under a chamber base pressure of 3× 10^−6^ mbar. A 15 nm zinc sulfide:silicon dioxide (ZnS:SiO_2_) capping layer is then added using a ZnS:SiO_2_ (80:20) target.

The deposited layers undergo thermal annealing at 250 °C for 10 min. The temperature is ramped up at 5 °Cs^−1^, while the cooling rate is 1 °Cs^−1^, to induce crystallization of the as-deposited PCM layer.

The line-shaped PCM cells are then defined using negative resist (AR-N 7520.07, ALLRESIST GmbH, Michendorf, Germany) and electron beam lithography, followed by RIE. After the etching process, a 100 nm aluminum oxide (Al_2_O_3_) layer is deposited via electron beam evaporation to encapsulate the devices.

### 2.2. RIE Process

To define the final PCM structure, the devices are etched using three different gas mixtures:

- CHF_3_:O_2_;
- H_2_:Ar;
- Ar.

The choice of RF power, the use of ICP, and the adjustment of gas ratios for each specific gas mixture are carefully optimized to achieve the desired etch profile and to ensure stable plasma ignition. The detailed parameters for each gas mixture are provided in the manuscript.

In order to assess the resulting device performance, initial resistance mapping and subsequent cycling tests are carried out using an aixMATRIX characterization tool from aixACCT Systems (Aachen, Germany), detailed in [29].

### 2.3. Measurement Setup

The aixMATRIX characterization system not only enables array-level measurements but also includes a wedge mode specifically designed for single-cell characterization. It operates at a sampling rate of 1 GS/s and incorporates a programmable current amplifier suitable for currents between 1 µA and 10 mA. Additionally, the system features a current compliance mechanism with a response time of 50 ns [19].

**Figure 2:**
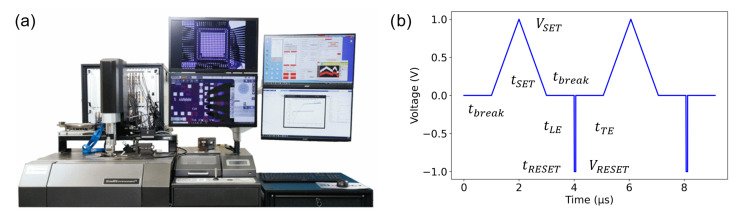
(**a**) aixMATRIX tester, reproduced with permission from [19]. (**b**) Waveform employed for characterization, consisting of two identical SET sweeps and two RESET pulses separated by a break interval. Device resistance is determined during the SET sweeps. Figure reproduced with permission from [19].

In this work, the wedge mode is utilized, which is capable of sequentially characterizing up to 556 individual PCM cells in one run. The measurement system, depicted in Figure 2a, is controlled via a Python-based interface (CASSINI) which coordinates interactions between the semi-automatic probe station and the electrical measurement setup [19]. This integrated solution supports automated probing and movement, enabling comprehensive characterization of targeted devices or entire samples without manual operation. This method is employed for initial resistance measurements after fabrication by providing the coordinates of a predefined set of devices on a chip to the software.

### 2.4. Measurement Algorithm

**Figure 3:**
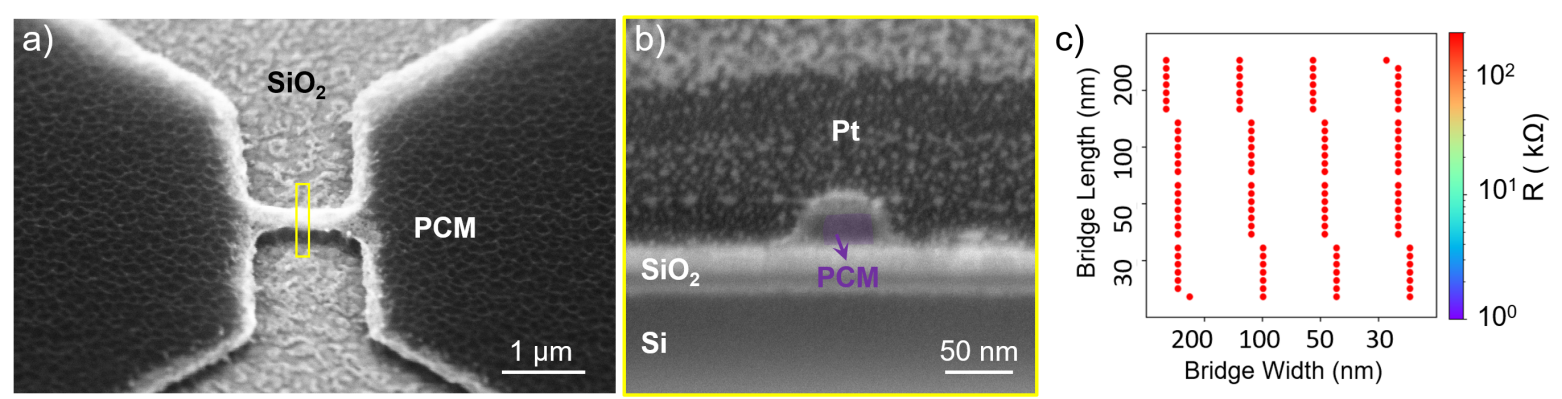
PCM cell etched with CHF_3_:O_2_ gas mixture: (**a**) Low-angle SEM view revealing etching residues. (**b**) Cross-sectional SEM image showing vertical PCM bridge profile (width: 30 nm). (**c**) Resistance map of a 10 mm × 10 mm sample after CHF_3_:O_2_ etching, indicating high initial resistances for all measured devices on the sample (red circles represent device locations and the color denotes resistance in kΩ).

**Figure 4:**
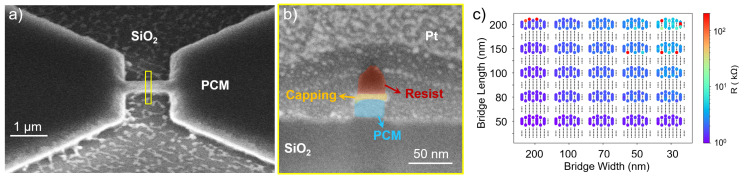
PCM device etched with H_2_:Ar gas mixture: (**a**) Tilted-view SEM image showing residue. (**b**) Cross-sectional SEM image depicting vertical PCM bridge profile (width: 30 nm). (**c**) Resistance map of a 25 mm × 25 mm sample after H_2_:Ar etching showing predominantly low initial resistances.

**Figure 5:**
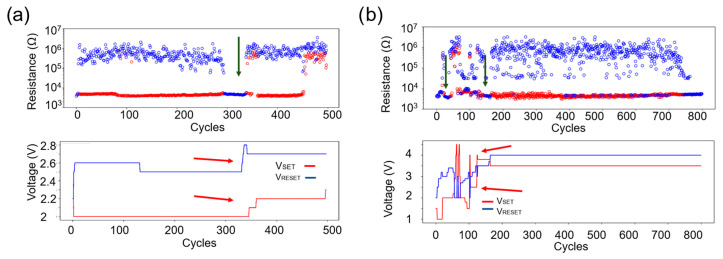
Cyclability test of two different devices etched with an H_2_:Ar gas mixture: (**a**) The upper graph displays the resistance states (HRS/LRS), with a green arrow indicating temporary non-functionality. The lower part of the image depicts SET/RESET voltages, with required adjustments marked by red arrows, illustrating instability after 300 cycles. (**b**) The upper graph presents the resistance states (HRS/LRS), with green arrows highlighting instabilities in the HRS/LRS ratio. The lower part of the image shows frequent voltage adjustments (red arrows). The device fails after approximately 700 cycles. In the upper graphs, red circles represent the resistance after SET, and blue circles represent the resistance after RESET.

**Figure 6:**
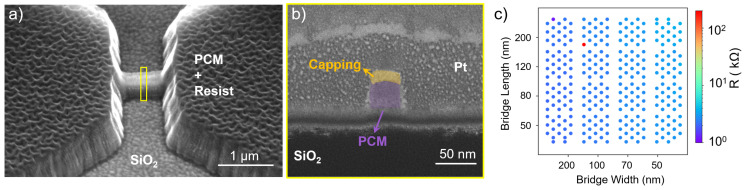
PCM cell etched with Ar gas: (**a**) Side-view SEM image confirming absence of residues. (**b**) Cross-sectional SEM image highlighting widening etch profile. (**c**) Initial resistance map of a 10 mm × 10 mm sample after Ar etching showing an ideal resistance range and high fabrication yield.

**Figure 7:**
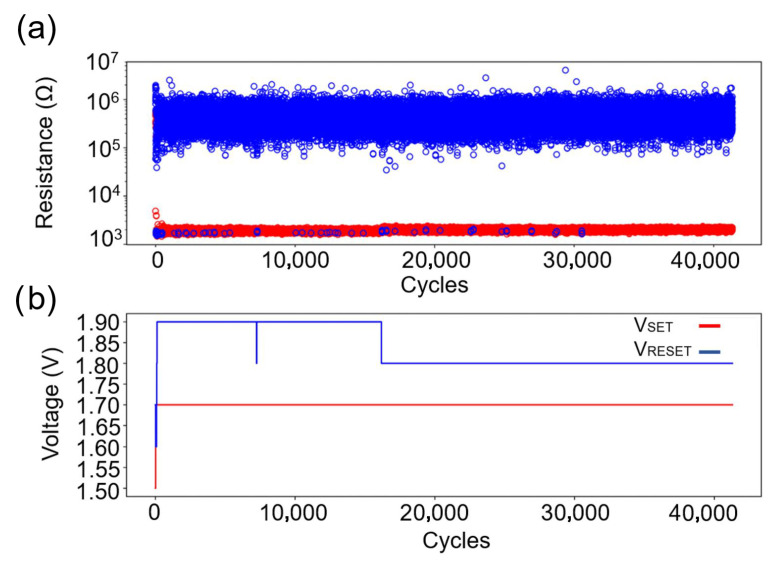
Cyclability test of Ar-etched PCM devices: (**a**) Stable HRS/LRS maintained over 40,000 cycles. Red circles represent the resistance after SET, and blue circles represent the resistance after RESET. (**b**) Minimal SET/RESET voltage variation, indicating reproducible switching behavior.

The algorithm used for cycling measurements employs the waveform shown in Figure 2b, in which the maximum voltages for the SET and RESET operations are systematically adjusted. During the SET operation, a voltage sweep is applied up to a maximum value of *V*_SET_ at a fixed sweep slew rate of 1 × 10^6^ V/s. The subsequent RESET operation comprises three phases: an initial leading edge of 10 ns (*t*_LE_), followed by maintaining a constant voltage *V*_RESET_ for 50 ns (*t*_RESET_), and, finally, a trailing edge of 1 ns (*t*_TE_). Furthermore, a break interval of 1 µs (*t*_break_) is inserted between consecutive pulses. Although PCM cells are inherently unipolar, opposite polarities are applied for the SET and RESET pulses. This method is intended to reduce ionic migration within the material, as highlighted in [30]. During read operations, the voltage amplitude is consistently held at ±1 V in both directions. During cycling measurements, the SET and RESET voltages are automatically regulated to maintain a stable ratio between the HRS and the LRS. This adaptive voltage adjustment enables continuous monitoring of the switching characteristics to verify their consistency over prolonged cycling. The measurement procedure is entirely automated, systematically iterating through each memory cell and identifying defective or non-functional cells. To further improve measurement precision, two calibration steps are performed. The first calibration occurs prior to cycling tests and is intended to determine optimal SET and RESET voltages, establishing suitable switching conditions. If a device fails to switch correctly during cycling, a second calibration step is conducted to re-adjust the SET and RESET voltages and restore functionality [19]. Moreover, to efficiently collect relevant data while minimizing measurement time, the maximum number of cycles per device is limited to a predefined value. This automated strategy significantly reduces manual intervention, improves measurement accuracy, and ensures robust and reliable testing. All electrical measurements are carried out in a two-probe configuration, where voltage pulses are applied through one probe and the resulting currents are measured through the other. Consequently, the reported resistances include the contact resistance. For a more detailed description of the measurement process, including the setup, cycling algorithm, and resistance evaluation methodology, the reader is referred to [19]. Moreover, the raw data and analysis scripts used to generate Figure 3, Figure 4, Figure 5, Figure 6 and Figure 7 are available in the Supplementary Materials.

## 3. Results and Discussion

### 3.1. CHF_3_:O_2_ Etching Process

The first tested RIE recipe utilizes CHF_3_ with a flow rate of 55 standard cubic centimeters per minute (sccm) and O_2_ at 5 sccm applied at a radio frequency (RF) power of 200 W, without inductively coupled plasma (ICP). The process is performed on four different GST compositions. The result is shown in Figure 3, with (a) illustrating residues or redeposited etching byproducts (likely GeF_x_, GeO_x_, TeF_x_, TeO_x_ [24]) around the PCM cell structure. This is undesirable as it requires an additional cleaning step to ensure that the GST bridge area and the regions between the GST pads are free of residues. Furthermore, the etch rate of the ZnS:SiO_2_ capping layer is very low (approximately 3–5 nm/min) under these conditions, requiring a longer etch duration. The etch rate is determined by measuring the step height between etched and masked regions using atomic force microscopy (AFM). However, scanning electron microscopy (SEM) analysis demonstrates the desired vertical etch profiles (Figure 3b). Vertical etch profiles are desired, as they preserve the intended dimensions defined by the mask. The initial resistances after etching with CHF_3_:O_2_ are notably high across all Ge-Sb-Te-based compounds, impeding cycling experiments due to difficulties in inducing sufficient current flow. Figure 3c provides an example resistance map of etched devices made of GST-225, clearly illustrating predominantly high resistance values > 50 kΩ across the sample. High resistances are likely related to compositional changes, such as Ge depletion. This is because Ge tends to form volatile GeF_4_ rapidly, whereas Sb and Te exhibit reduced etch rates due to the formation of nonvolatile compounds such as TeO_2_, TeO_x_F_y_, and SbF_5_, as reported in [23,24,31], both within the bridge volume and along the bridge sidewalls. This interpretation, however, requires further experimental validation to be confirmed.

### 3.2. H_2_:Ar Etching Process

Given the low etch rate of the capping layer and the high initial resistance of the samples reactively etched in the CHF_3_:O_2_ environment, the alternative of more physical etching is employed using an H_2_ (48 sccm) and Ar (12 sccm) gas mixture with ICP power of 250 W and RF power of 25 W. The use of the H_2_:Ar gas mixture results in an etching rate of 20 nm/min for the capping layer, as determined by measuring the step height between the etched and masked regions using atomic force microscopy, effectively resolving the low etch rate issue observed with CHF_3_:O_2_.

However, residues originating from the sputtered layer or redeposited nonvolatile compounds are observed on the SiO_2_ substrate after etching (Figure 4a). Therefore, an additional cleaning step is required to ensure a clean area between the large pads and in the vicinity of the bridge. Despite this, the process demonstrates high selectivity, resulting in minimal resist consumption and the desired vertical etch profiles, as shown in Figure 4b. Initial resistance measurements show significantly reduced resistance compared to the previous etching approach, enabling further cycling characterization (Figure 4c). This reduction is tentatively attributed to the action of hydrogen in the H_2_:Ar plasma. Hydrogen has been shown to react preferentially with Te and Sb before Ge (Te > Sb > Ge), forming volatile hydrides [32]. The resulting depletion of Te and Sb, and the corresponding shift in the composition of the alloy within the bridge volume, could be one possible reason for the lower initial resistance observed. Additional experiments will be required to confirm this hypothesis and to clarify the extent to which compositional modifications influence device behavior. Figure 4c also shows several devices with high initial resistance and a clear resistance gradient correlated with the device dimensions. The high resistances may arise from imperfect contact between the GST cell and the bottom electrode, from non-ideal contact between the probe tips and the device pads during measurement, or from a disconnected bridge between the pads. The different resistance patterns observed in Figure 3c and Figure 4c are attributed to the number of devices measured in each case. Initially, a quick measurement on a smaller subset of devices (Figure 3c) is performed to evaluate whether the devices exhibit resistances in the desired resistance range. If promising results are observed, a more extensive and time-consuming measurement is carried out on a larger number of devices (Figure 4c).

Despite the lower initial resistance values, the cycling tests in Figure 5a,b reveal an unstable LRS and HRS, requiring SET and RESET voltage adjustments after a relatively low number of switching cycles to maintain resistance stability. The measured devices exhibit rapid degradation, failing to switch states reliably. Cells alternate between temporary non-functionality and eventually becoming stuck in either the LRS or HRS, indicating limited endurance under this etching regimen. The rapid degradation observed under this etching regime could again be attributed to stoichiometric shifts, as explained above. Such compositional changes could impair phase-change dynamics, leading to instability in the HRS and LRS values and to accelerated failure during cycling. However, additional investigations will be required to confirm this hypothesis and to quantify the extent to which this compositional change contributes to the limited endurance.

### 3.3. Ar Etching Process

To enhance the device performance and mitigate issues identified with previous gas mixtures, a purely physical etching process using Ar gas is tested. Etching is conducted with an Ar gas flow rate of 20 sccm, ICP power of 250 W, and RF power of 25 W. Initially, the ZnS:SiO_2_ capping layer is etched using the H_2_:Ar recipe for 45 seconds, stopping just above the phase-change layer. This is followed by Ar etching that results in residue-free and clean PCM structures (Figure 6a) exhibiting a slightly tapered etch profile (wider at the base), potentially resulting in a minor loss of critical dimension (Figure 6b).

The initial resistance measurements demonstrate resistances in the range of 1–5 kΩ (Figure 6c). This range has proven to be promising for reliable switching characterization, because the required currents for inducing sufficient Joule heating for the device dimensions used in this work are typically currents up to 5 mA. Very high resistances, such as the results of the CHF_3_:O_2_ etching recipe, would require very high voltages to drive sufficient current through the device.

Conversely, very low resistances, as observed in devices etched with H_2_:Ar, are problematic, since the process is governed by Joule heating and the device does not heat up sufficiently for a RESET operation. Therefore, the observed resistance range of 1–5 kΩ is considered ideal, as the voltage range of the used setup corresponds to the required voltages for inducing the RESET current. Moreover, this etching process results in a high yield (99.5%), making it suitable for scalable device fabrication (Figure 6c). The single device exhibiting a higher resistance value may be attributed to a poor electrical connection to the bottom contact or to a local defect in the bridge area. Cyclability tests indicate superior stability, with stable resistance states (HRS and LRS) maintained consistently over 40,000 cycles without frequent voltage adjustments (Figure 7). This behavior is observed across all compositions studied in this work, including AIST, GST-124, GST-147, and GST-225.

## 4. Conclusions

This study systematically investigated the impact of three different RIE gas mixtures, CHF_3_:O_2_, H_2_:Ar, and Ar, on the fabrication, initial resistance, and cycling performance of line-type phase-change memory devices. The CHF_3_:O_2_ etching resulted in high initial resistances that prevented any switching events, most likely due to compositional alterations arising either from the rapid formation of volatile GeF_4_ or from the formation of nonvolatile species such as TeO_2_, TeO_x_F_y_, and SbF_5_, as reported in [23,24,31], within the bridge volume. Shifting to a physically oriented H_2_:Ar etching recipe significantly reduced the initial resistance. However, it suffered from cycling instability, limiting reliable cycling performance to less than a few hundred cycles, which may be related to Sb/Te depletion effects, as discussed in prior work on the hydrogen plasma etching of GST thin films [32]. A subsequent transition to a purely physical etching process using Ar gas effectively mitigated these drawbacks, achieving appropriate initial resistances, high device yield (99.5%), and robust and stable cycling exceeding 40,000 cycles. Our discussion connects the observed electrical behavior to plausible plasma chemistry pathways, which are supported by previous studies on comparable compositions. While direct compositional analyses (e.g., TEM, XPS, EDX) were not performed in this work, future studies could incorporate them to confirm stoichiometric changes and residues and to strengthen correlations with device performance. Moreover, since all GST films in this study were deposited under constant sputtering conditions and capped with ZnS:SiO_2_, we conclude that the observed device performance can be attributed primarily to the applied etching chemistry on the GST compounds, with only minor contributions from possible small variations in film composition or density. Automated cycling testing via the aixMATRIX system facilitated comprehensive characterization and optimization, helping to reveal that the Ar etching strategy is highly advantageous for stable, reproducible, and reliable PCM device fabrication.

## Data Availability

The original contributions presented in this study are included in the article and its Supplementary Materials. Further inquiries can be directed to the corresponding author.

## Supplementary Materials

The following supporting information can be downloaded at: https://www.mdpi.com/article/10.3390/ma18204681/s1, File S1: Data_PCM—Folder containing raw and processed measurement data corresponding to Figure 3, Figure 4, Figure 5, Figure 6 and Figure 7, organized by figure number (Figure_3_c–Figure_7). This includes CSV datasets, PKL files, and generated plots for each figure. File S2: get_data_PCM.py—Python script used for data access, processing, and reproduction of plots shown in Figure 3, Figure 4, Figure 5, Figure 6 and Figure 7. File S3: Read_me.txt—Documentation describing the folder hierarchy, data structure, and instructions for reproducing the analysis.

## Abbreviations

The following abbreviations are used in this manuscript:

PCM	Phase-Change Memory
GST	Ge_x_Sb_y_Te_z_ (Germanium–Antimony–Tellurium)
AIST	Ag_4_In_3_Sb_67_Te_26_ (Silver–Indium–Antimony–Tellurium)
RIE	Reactive Ion Etching
CPW	Coplanar Waveguide
RF	Radio Frequency
ICP	Inductively Coupled Plasma
HRS	High-Resistance State
LRS	Low-Resistance State
SEM	Scanning Electron Microscopy
AFM	Atomic Force Microscopy
sccm	Standard Cubic Centimeters per Minute
Pt	Platinum
Ti	Titanium
Al_2_O_3_	Aluminum Oxide
ZnS:SiO_2_	Zinc Sulfide–Silicon Dioxide Composite
SET	Operation that switches PCM to crystalline (low-resistance) state
RESET	Operation that switches PCM to amorphous (high-resistance) state
GS/s	Giga-Samples per Second
CMOS	Complementary Metal–Oxide–Semiconductor
CD	Critical Dimension
aixMATRIX	Automated Test System from aixACCT Systems
CASSINI	Python-Based Software Interface for Electrical Characterization
