# Supplementary figures and images for: The Influence of Reactive Ion Etching Chemistry on the Initial Resistance and Cycling Stability of Line-Type (Bridge) Phase-Change Memory Devices
Source: Materials (Basel). 2025 Oct 12;18(20):4681. doi: 10.3390/ma18204681 (PMC12565918; doi:10.3390/ma18204681)

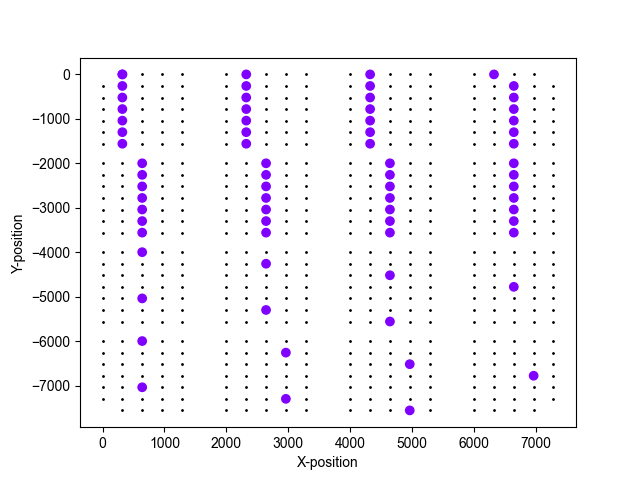

Supplement: Supplementary file 1 [file materials-18-04681-s001.zip › File S1-Data_PCM/Figure 3_c/AIXCT_02/Data/plot/Init_res.png]

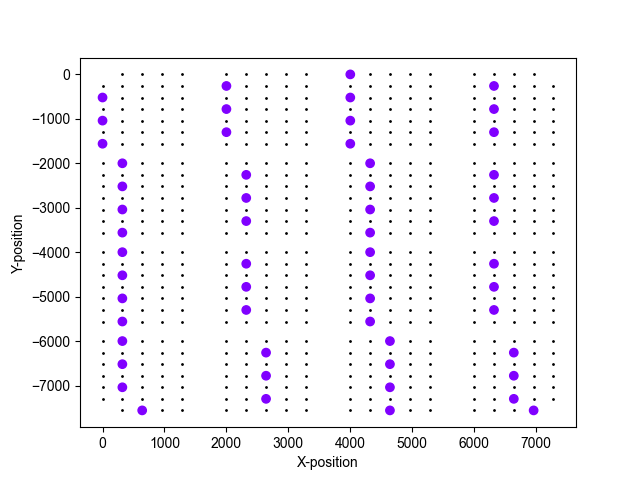

Supplement: Supplementary file 1 [file materials-18-04681-s001.zip › File S1-Data_PCM/Figure 3_c/AIXCT_03/Data/plot/Init_res.png]

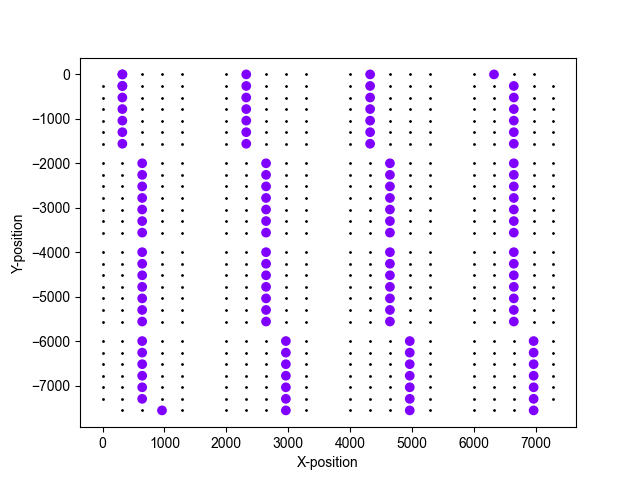

Supplement: Supplementary file 1 [file materials-18-04681-s001.zip › File S1-Data_PCM/Figure 3_c/AIXCT_04/Data/plot/Init_res.png]

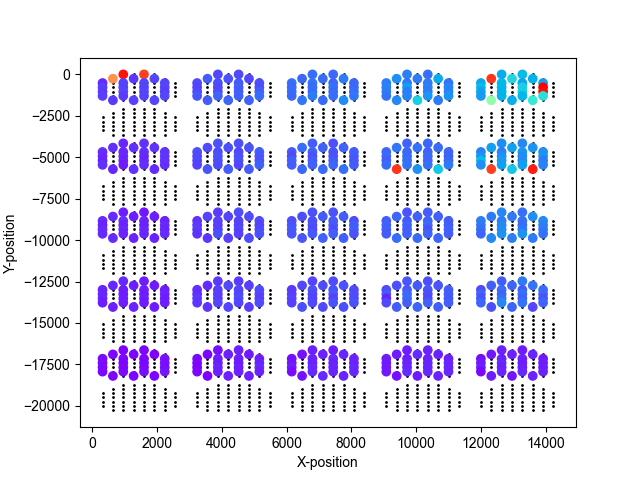

Supplement: Supplementary file 1 [file materials-18-04681-s001.zip › File S1-Data_PCM/Figure 4_c/Data/plot/Init_res.png]

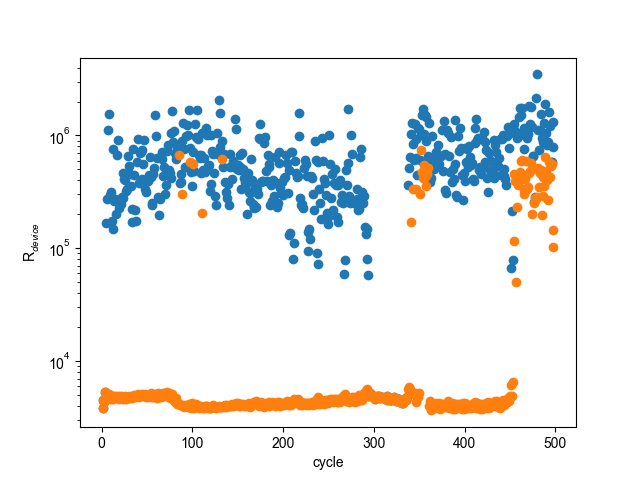

Supplement: Supplementary file 1 [file materials-18-04681-s001.zip › File S1-Data_PCM/Figure 5_a/Data/plot/plot_resistance.png]

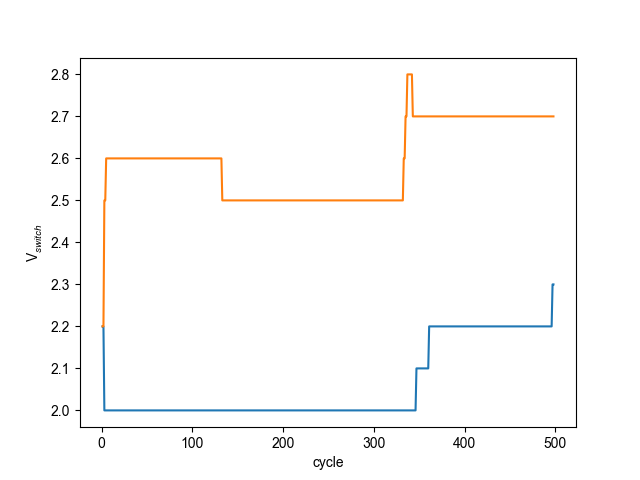

Supplement: Supplementary file 1 [file materials-18-04681-s001.zip › File S1-Data_PCM/Figure 5_a/Data/plot/plot_voltage.png]

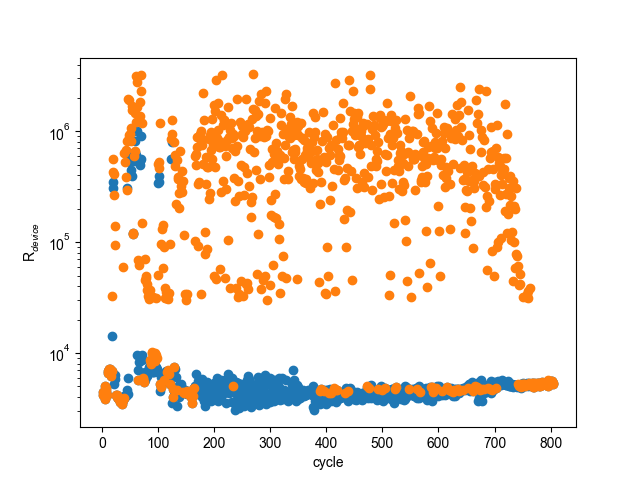

Supplement: Supplementary file 1 [file materials-18-04681-s001.zip › File S1-Data_PCM/Figure 5_b/Data/plot/plot_resistance.png]

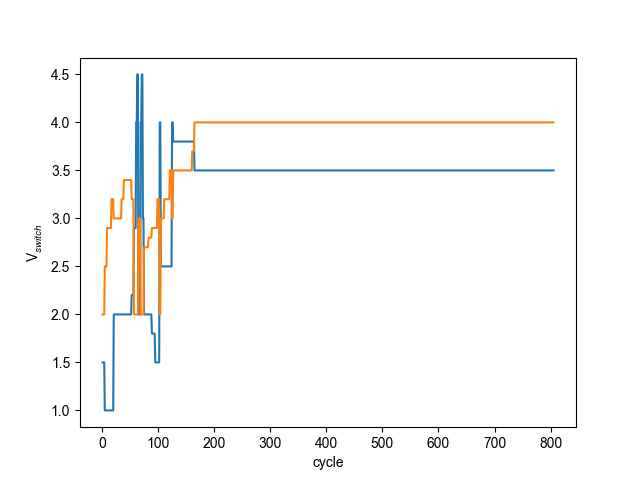

Supplement: Supplementary file 1 [file materials-18-04681-s001.zip › File S1-Data_PCM/Figure 5_b/Data/plot/plot_voltage.png]

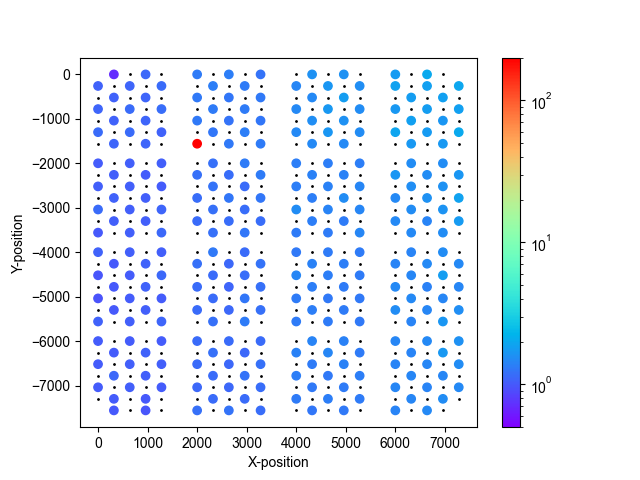

Supplement: Supplementary file 1 [file materials-18-04681-s001.zip › File S1-Data_PCM/Figure 6_c/Data/plot/Init_res.png]

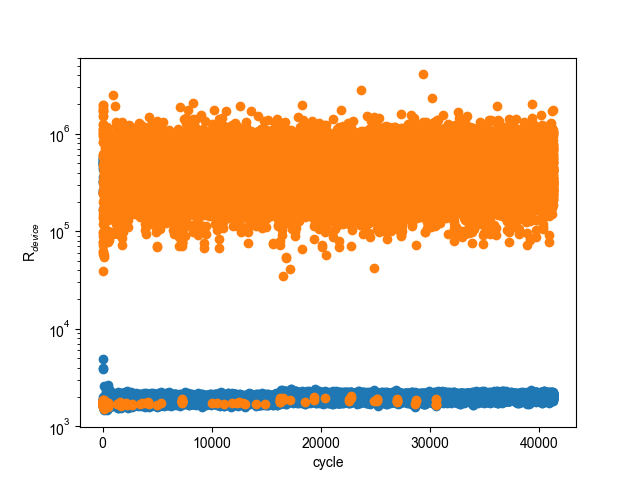

Supplement: Supplementary file 1 [file materials-18-04681-s001.zip › File S1-Data_PCM/Figure 7/Data/plot/plot_resistance.png]

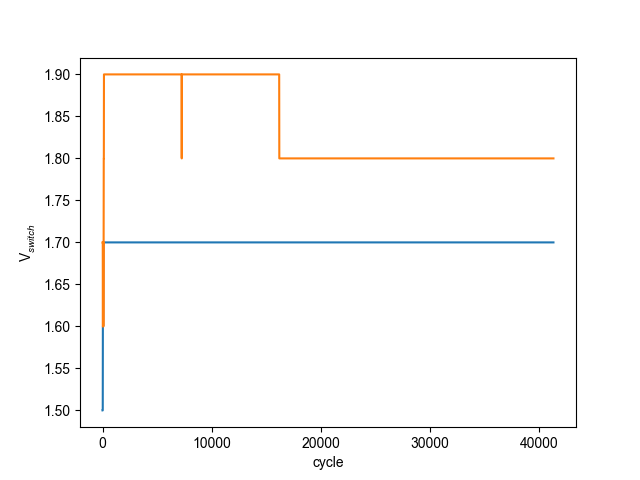

Supplement: Supplementary file 1 [file materials-18-04681-s001.zip › File S1-Data_PCM/Figure 7/Data/plot/plot_voltage.png]
